# Glutamate: Safe and Adequate Intake Levels for Infants—*Should Breast Milk Be Taken Off the Market?*

**DOI:** 10.3390/foods15091530

**Published:** 2026-04-28

**Authors:** Anita Hartog, Hans Verhagen

**Affiliations:** 1 Food for Health Consultancy, 6986BK Angerlo, The Netherlands; 2 Food Safety & Nutrition Consultancy, 3703EE Zeist, The Netherlands; 3 School of Biomedical Sciences, Faculty of Life and Health Sciences, Ulster University, Coleraine BT52 1SA, UK; 4 National Food Institute, Technical University of Denmark, Kemitorvet 201, 2800 Kgs. Lyngby, Denmark

**Keywords:** context-driven toxicological risk assessment, acceptable daily intake, health-based guidance values, risks, benefits, glutamate, human milk oligosaccharides, breast milk, vitamin C, iodine

## Abstract

Toxicological risk assessment of food ingredients has traditionally relied on identifying a no-observed-adverse-effect level (NOAEL) or benchmark dose (BMD), followed by the application of default uncertainty factors (UFs) to derive health-based guidance values (HBGVs) such as the acceptable daily intake (ADI). While effective for conventional food additives, this approach may be inappropriate for nutrients and intrinsic food components with established physiological functions. This paper critically explores these limitations using free glutamate as a central example, alongside additional cases relevant to infant nutrition, including vitamin C, iodine, and human milk oligosaccharides (HMOs). Data on free glutamate in human milk show that breastfed infants habitually ingest amounts far exceeding additive-based ADIs without adverse effects, underscoring the limitations of applying default uncertainty factors and classical toxicological paradigms to endogenous nutrients. Comparable considerations apply to protein hydrolysates and amino acid-based infant formulas evaluated by EFSA, where growth, tolerance, and compositional suitability are integral to safety assessment. Overall, nutrient safety evaluation requires an integrative, physiology-informed framework that incorporates realistic exposure, developmental stage, and metabolic competence. Breast milk provides a biologically relevant reference, supporting a proportionate and science-based application of toxicological principles in infant nutrition.

## 1. Introduction to Toxicological Risk Assessment of Food Ingredients

**Figure 1:**
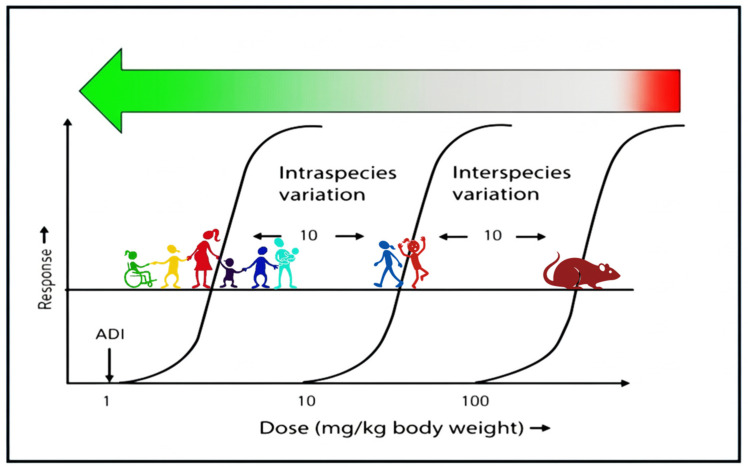
Concept of deriving an acceptable daily intake (ADI) from a point of departure (NOAEL or BMDL). Effective (adverse) dose levels are at the high end (in red), whereas lower dose levels are without effects (in green). A safety factor of, e.g., 100 (10 for interspecies differences and 10 for intraspecies differences) is typically used to calculate an ADI that is without effects in humans. Dose levels in the figure are for illustrative purposes only. Figure adapted from Logue et al. [2].

Toxicological risk assessment provides the scientific basis for establishing safe levels of exposure to food ingredients, including food additives, processing aids, and other intentionally added substances. Its central objective is to determine whether, and under what conditions of use, a substance may pose a risk to human health. Within food safety regulation, this process has evolved into a structured and internationally harmonised paradigm, most prominently expressed through the derivation of health-based guidance values (HBGVs) such as the acceptable daily intake (ADI) [1,2,3]. At the core of toxicological risk assessment lies the distinction between *hazard* and *risk*. A hazard represents the intrinsic potential of a substance to cause adverse effects, whereas risk depends on both hazard and the magnitude, frequency, and duration of human exposure. Virtually any substance, including essential nutrients or naturally occurring food constituents, can become hazardous at sufficiently high intakes. Toxicological risk assessment therefore seeks not to identify “safe” or “unsafe” substances in absolute terms, but to define exposure levels that are unlikely to result in adverse health effects in humans (Figure 1).

The classical framework for food-additive risk assessment comprises four interrelated steps: hazard identification, hazard characterisation, exposure assessment, and risk characterisation. Among these, hazard characterisation is pivotal for establishing quantitative reference points that form the basis for regulatory decision making. Traditionally, this step relies on controlled toxicological studies, most often conducted in experimental animals, designed to identify dose–response relationships for adverse effects following repeated oral exposure. Historically, the primary reference point used in food-additive risk assessment has been the no-observed-adverse-effect level (NOAEL), defined as the highest tested dose at which no biologically or toxicologically relevant adverse effects are observed. More recently, benchmark dose (BMD) modelling has gained increasing acceptance as an alternative and often preferable approach. Rather than relying on a single experimental dose, the BMD approach uses the full dose–response dataset to estimate the dose associated with a predefined small increase in response (e.g., 5 or 10%), together with its lower confidence limit (BMDL). Both NOAELs and BMDLs serve as “points of departure” for deriving HBGVs (Figure 1) [4,5].

To translate these experimental reference points into values considered protective for humans, uncertainty (or safety) factors are applied. The default approach for food additives typically uses a composite uncertainty factor (UF) of 100, comprising a factor of 10 to account for interspecies differences between experimental animals and humans, and an additional factor of 10 to account for variability within the human population. This convention reflects both biological uncertainty and variability, including differences in toxicokinetics and toxicodynamics among individuals. The resulting ADI for humans was originally developed by the Joint FAO/WHO Expert Committee on Food Additives (JECFA) and defined as “an estimate of the amount of a food additive, expressed on a body weight basis, that can be ingested daily over a lifetime without appreciable health risk” [6].

The robustness of the ADI concept rests on its intentionally conservative nature. ADIs are not toxicity thresholds but protective reference values, designed to remain well below doses associated with adverse effects, even for sensitive subpopulations. Importantly, consumption below the ADI does not imply exposure to a harmful level, nor does occasional exceedance automatically indicate a health risk. Rather, the ADI functions as a benchmark for long-term risk management and regulatory decision-making.

While the NOAEL/BMD-UF paradigm has proven effective for classical food additives, its application becomes more complex for substances that also occur naturally in the diet or fulfil physiological roles, such as (essential) nutrients or nutrient-like compounds. In such cases, background dietary exposure may already approach or exceed levels that would be considered acceptable under a default food-additive framework. This tension has been highlighted in assessments of substances such as amino acids and proteins, minerals, or vitamins, where toxicological data must be interpreted alongside nutritional considerations and established patterns of human intake [7,8,9,10].

For nutrients, risk assessment has historically focused on defining tolerable upper intake levels (ULs), which represent the maximum total chronic intake from all sources unlikely to pose a risk of adverse effects. Although ULs and ADIs are conceptually similar, their derivation may differ in practice because nutrients are subject to homeostatic control and are essential within a certain intake range. Recent regulatory discussions emphasise the need for harmonised approaches when substances are assessed both as regulated products and as nutrients, including transparent consideration of total exposure and biological context [3,11].

Beyond classical hazard-based assessment, toxicological risk assessment increasingly interfaces with broader evaluative frameworks, such as risk–benefit assessment, particularly for micronutrients and bioactive substances. For foods and food constituents that can confer both health benefits and risks, risk–benefit assessment offers a structured way of integrating favourable and adverse health outcomes within a single paradigm, rather than treating them in isolation. In the context of nutrients and nutrient-like substances, this implies explicitly weighing the consequences of both excessive and insufficient intakes, and considering how shifts in exposure scenarios may alter the overall balance of health impact across different population groups. These approaches recognise that both insufficient and excessive intakes may carry health risks, and that optimal intake ranges cannot always be defined by toxicity alone. While such integrated approaches are not standard for food additives, they underscore the importance of interpreting toxicological reference values within the wider context of human nutrition and exposure patterns [12,13,14,15,16,17,18,19,20,21].

In summary, toxicological risk assessment of food ingredients relies on a well-established methodological foundation centred on experimentally derived reference points, UFs, and the derivation of ADIs. This framework has delivered a high level of consumer protection for decades. At the same time, ongoing scientific and regulatory developments continue to refine the application of these principles, particularly for substances at the interface of toxicology and nutrition. A clear understanding of the underlying concepts, NOAELs and BMDs, UFs, and HBGVs is therefore essential for interpreting food safety assessments and their implications for public health.

For clarity, a list of abbreviations used in this review is provided below, before the reference list.

## 2. Conceptual Background: When ADIs Work and When They Do Not

### 2.1. Vitamin C and the Limits of Classical Risk Assessment

Vitamin C (ascorbic acid) provides a clear and instructive example of why the classical toxicological risk assessment paradigm, developed primarily for food additives, does not translate straightforwardly to nutrients. As an essential micronutrient, vitamin C is required for normal physiological function, acting as a cofactor in collagen and carnitine synthesis, in hormone and neurotransmitter biosynthesis, in epigenetic regulation, and as a water-soluble antioxidant, yet it can also cause adverse effects at high intakes [22]. This dual role illustrates inherent limitations of applying the standard NOAEL/BMD-UF approach in a manner equivalent to non-essential chemicals. In recognition of this complexity, the European Food Safety Authority’s Scientific Panel on Dietetic Products, Nutrition and Allergies (NDA) has published specific guidance on deriving ULs for vitamins and minerals, including vitamin C. These ULs are distinct from ADIs for food additives and are rooted in both toxicological and nutritional considerations [3,23].

For vitamin C, the hazard characterisation underpinning the UL is based largely on controlled human data rather than animal studies, as many laboratory animal species synthesise ascorbic acid endogenously and therefore do not serve as reliable models for human exposure. Human supplementation studies have consistently shown that the primary adverse effect at high intakes is gastrointestinal disturbance, particularly osmotic diarrhoea and related symptoms such as cramps and nausea, effects that are typically mild, rapidly reversible upon dose reduction, and not associated with systemic toxicity.

In its 2004 opinion on vitamin C, the European Food Safety Authority (EFSA) NDA Panel (Panel on Dietetic Products, Nutrition and Allergies) identified a lowest observed adverse effect level (LOAEL) of about 3 g per day in adults based on the onset of diarrhoea and associated gastrointestinal effects in supplementation studies. A clear NOAEL could not be established consistently across available studies, reflecting variability in individual tolerance and study design. On this basis, EFSA concluded that the available data did not support the derivation of a sufficiently robust numerical UL for vitamin C in the general population and therefore refrained from setting one [24]. By contrast, for vitamin C as a food additive, the EFSA Panel on Food Ingredients and Packaging noted that supplemental intakes of up to 1000 mg/day, in addition to the usual dietary intake, were not associated with adverse gastrointestinal effects and considered this level unlikely to pose a risk in the general adult population [25]. In addition, the US Institute of Medicine (IOM) established a UL of 2 g/day, derived from the LOAEL of 3 g/day [26]. Taken together, these evaluations illustrate the complexity of a food ingredient that is also an essential nutrient and regulated product.

This derivation illustrates several departures from the classical food-additive risk assessment paradigm:

1. **Human data drive the reference point**. Since the critical effects are observed directly in human subjects, there is no need for an interspecies extrapolation factor. This eliminates a default 10-fold UF and grounds the UL in human biological responses.
2. **The UF for intraspecies variability is modest**. Because the observed effect (diarrhoea) is a relatively mild, non-systemic response and because high-dose supplementation studies allow direct observation of dose–response, the EFSA NDA Panel applied a small factor to bridge remaining uncertainty rather than the default 10-fold factor typically used for food additives.
3. **The point of departure is expressed as an absolute daily intake**. Unlike food additives, where reference points are usually normalised to body weight (e.g., mg/kg bw/day), upper intake levels for nutrients are often expressed in absolute daily amounts (e.g., grams per day). This reflects both the nature of the effect and the practical context of nutritional recommendations, where body weight scaling is less informative for gastrointestinal responses.

The vitamin C example underscores that, for nutrients, adverse effects at high intakes must be interpreted in the context of physiological function, homeostatic regulation, background exposure, and the severity and reversibility of effects. Mild gastrointestinal symptoms differ qualitatively and quantitatively from toxicological endpoints typically used to regulate food additives (such as organ toxicity or carcinogenicity). Consequently, derivation of HBGVs for nutrients requires a nuanced application of toxicological principles, integrating human data, nutritional requirements, and biological plausibility, consistent with international guidance such as EFSA’s UL framework.

This case highlights why risk assessment frameworks for nutrients and food additives, while conceptually related, cannot be treated as interchangeable. When a substance is both a nutrient and potentially harmful at high intakes, risk assessment must balance nutritional adequacy with toxicity risk, ensuring that reference values support health across a realistic range of intakes without imposing undue conservatism.

### 2.2. Illustrative Example: Iodine and the Narrow Margin Between Benefit and Risk

Iodine provides a complementary example to vitamin C, illustrating a different, but equally important, limitation of applying the classical food-additive risk assessment paradigm to nutrients. Whereas vitamin C demonstrates how default UFs can lead to unrealistically low “safe” levels, iodine highlights the challenge of nutrients with a narrow margin between adequate and excessive intake, and strong physiological feedback mechanisms.

Iodine is an essential trace element required for the synthesis of thyroid hormones, which are critical for growth, neurodevelopment, and metabolic regulation [27,28]. Both iodine deficiency and iodine excess are associated with adverse health outcomes, particularly involving thyroid function [29]. This U-shaped intake–response relationship is characteristic of many micronutrients and fundamentally differs from the monotonic dose–response relationships typically assumed for food additives.

The derivation of the UL for iodine, as with vitamin C, has relied heavily on human data, including observational studies and controlled interventions, rather than on animal toxicity studies alone. Excess iodine intake has been associated with a range of thyroid-related effects, including elevated thyroid-stimulating hormone (TSH), hypothyroidism, hyperthyroidism, and autoimmune thyroiditis. Importantly, these effects are mediated through well-characterised physiological mechanisms, including the acute Wolff–Chaikoff effect and failure to escape from this iodine-induced inhibition in susceptible individuals, as well as Jod-Basedow-type iodine-induced hyperthyroidism [29,30].

In its evaluations, EFSA identified thyroid dysfunction, rather than overt toxicity or irreversible organ damage, as the critical adverse effect for iodine excess. The point of departure for establishing the upper intake level was derived from human studies indicating biochemical changes in TSH and TSH response to thyrotropin-releasing hormone at habitual intakes in the range of 1.7–1.8 mg of iodine per day in adults, without associated clinical hypothyroidism. Applying a UF of about 3 to account for interindividual variability and sensitive subgroups, the EFSA established a UL of 600 µg/day for adults, including pregnant and lactating women [31].

This derivation again departs in important ways from the classical food-additive risk assessment approach:

1. **Adverse effects reflect dysregulation rather than toxicity**. The critical effects used to define the upper level for iodine are functional disturbances of endocrine regulation, not direct toxic injury. These effects may be reversible but can be clinically significant, particularly in vulnerable groups.
2. **Human variability dominates risk characterisation**. Sensitivity to excess iodine varies widely between individuals and populations, depending on baseline iodine status, age, pregnancy, pre-existing thyroid disease, and genetic predisposition. This variability cannot be captured adequately by default UFs derived from animal-to-human extrapolation.
3. **Background intake is central to risk assessment**. Unlike food additives, iodine intake from natural dietary sources and fortified foods contributes substantially to total exposure. Risk characterisation therefore requires explicit consideration of total intake from all sources, not merely the incremental contribution of a regulated use.
4. **Application of a classical additive approach would be misleading**. EFSA has set an Adequate Intake at 150 µg/day for adults [31]. If one were to apply a standard NOAEL/LOAEL-based approach with a default UF of 100 to the human reference point for iodine, the resulting “acceptable” intake would fall far below the intake required to ensure adequate thyroid hormone production (1.7–1.8 mg/100 leads to a mere …17–18 µg/day, whereas the established AI is 600 µg/day). Such an outcome would paradoxically increase the risk of deficiency-related disorders.

The iodine example demonstrates that, for some nutrients, risk assessment is less about identifying a large margin of safety below a toxic threshold and more about managing a narrow safe range of intake. In this context, the objective of the health-based guidance value is not to separate “safe” from “unsafe” exposure in absolute terms, but to support risk management decisions that minimise the likelihood of both deficiency and excess across diverse population groups.

Together, the examples of vitamin C and iodine illustrate two distinct ways in which nutrients challenge the classical toxicological risk assessment paradigm. Vitamin C shows that default UFs can generate biologically meaningless outcomes when applied to mild, local, and reversible effects. Iodine shows that even when adverse effects are clearly defined, the presence of essentiality, homeostatic control, and population variability necessitates a fundamentally different interpretation of reference points and safety margins. These cases underscore why nutrients require tailored risk assessment approaches that integrate toxicology with human physiology and nutrition science.

## 3. Glutamate and the Inapplicability of Additive-Based Risk Assessment to Macronutrient-like Substances

Glutamate represents a third, distinct category in which the classical toxicological risk-assessment paradigm becomes problematic: it functions both as a food additive and as an abundant dietary constituent with physiological roles, consumed in gram quantities as part of a normal diet. Unlike vitamin C or iodine, glutamate is a non-essential amino acid and a major metabolic substrate, particularly for intestinal and neural tissues. Its case illustrates how applying a food-additive framework without considering nutritional context and toxicokinetics can yield biologically implausible conclusions.

From a regulatory-science perspective, it is important to recognise that EFSA’s 2017 re-evaluation of glutamic acid and glutamates was conducted under the general framework for food additive assessment, which emphasises transparent dose–response characterisation, benchmark modelling where feasible, and the application of uncertainty factors to the most appropriate point of departure, whether based on animal or human data. EFSA’s role is to provide independent risk assessment to the European Commission and Member States, who then make the risk management decisions, and its opinions are intentionally cautious in order to support a high level of consumer protection. In this context, the numerical group ADI of 30 mg/kg bw per day can be understood as a consistent application of this framework, rather than as a dismissal of nutritional or physiological evidence. The tension highlighted in this Viewpoint therefore lies not in a failure of EFSA as an institution, but in the structural limitations of applying an additive-oriented paradigm to substances that function as nutrient-like metabolic substrates and are habitually consumed at levels close to or above such guidance values.

Glutamate is naturally present as one of the most abundant amino acids in dietary proteins and also occurs in free form in many commonly consumed foods, such as tomatoes, cheeses, fermented products, and human milk [32]. Total (protein-bound plus free) glutamate intakes are in the gram per day range and, when expressed per kg of body weight, reach approximately 80–300 mg/kg per day in many age groups, i.e., clearly above the EFSA group ADI of 30 mg/kg per day, which, however, applies to added free glutamate. By contrast, free glutamate intakes are substantially lower than total glutamate intakes and generally remain below this group ADI in most age groups, with the notable exception being breastfed infants and toddlers for whom estimated free glutamate intakes from, e.g., human milk, exceed 30 mg/kg body weight per day. As a result, ADIs set by classical toxicology can fall below habitual intakes, including in infants and toddlers, which is conceptually problematic for a substance that is an integral component of normal metabolism and diet rather than a trace xenobiotic [9,33,34].

A key reason for this mismatch lies in toxicokinetics. Orally ingested glutamate is extensively metabolised in the gastrointestinal tract, where it serves as a primary energy source for enterocytes [35]. As a result, systemic exposure following dietary intake is limited, and plasma glutamate concentrations remain tightly regulated across a wide range of intakes. Only under artificial exposure scenarios, such as large bolus doses of free glutamate administered in water or capsules, are transient increases in plasma glutamate observed, conditions that do not reflect realistic dietary consumption. The classical risk assessment paradigm does not adequately account for such route- and matrix-dependent kinetics.

In addition, glutamate is subject to strict physiological regulation, particularly with respect to central nervous system exposure. The blood–brain barrier expresses high capacity transport systems that limit glutamate influx and promote efflux, so brain glutamate homeostasis depends mainly on capacity transport systems that limit glutamate influx and promote efflux, so brain glutamate homeostasis depends mainly on local metabolism and synaptic cycling rather than on circulating levels [36]. Consequently, neurotoxic effects reported in experimental models using parenteral injections or extreme bolus doses cannot and should not be directly extrapolated to humans consuming (free) glutamate as part of ordinary diets, where plasma concentrations never approach the thresholds associated with excitotoxic damage in rodents. Taken together, these kinetic and regulatory features mean that adverse neurotoxic effects observed in specific experimental models or life stages cannot be straightforwardly extrapolated to the general population under normal dietary conditions.

If the default food-additive approach is applied mechanically, selecting a NOAEL from a sensitive animal study and applying a 100-fold UF, the resulting ADI for free glutamate may be lower than the intake from exclusively human milk feeding or ordinary mixed diets. Such outcomes illustrate a fundamental limitation of the paradigm: it implicitly assumes that the substance under evaluation is not an integral part of normal human metabolism and diet and that background exposure is negligible relative to the point of departure. In contrast to vitamin C (where mild, reversible gastrointestinal endpoints challenge the use of large UFs) and iodine (where a narrow safe range is shaped by endocrine regulation), glutamate shows that for substances with high background exposure, extensive first-pass metabolism, and central metabolic roles, the very concept of a numerically restrictive ADI may lose practical relevance.

Rather than indicating a genuine safety concern, excessively low ADIs for free glutamate reflect the inappropriate application of a hazard-based framework to a nutrient-like substance. In such cases, risk assessment must place greater emphasis on human kinetic data, realistic dietary exposure, and biological function, and must distinguish between theoretical hazard and actual risk under conditions of normal consumption.

Concerns regarding the appropriateness of applying default toxicological UFs to glutamate are not new and have been raised explicitly in several authoritative risk assessments and scientific commentaries. In its 2017 scientific opinion [37], EFSA reviewed the available toxicological and human data on glutamic acid and glutamates and concluded that adverse effects were observed only at very high bolus doses, often administered in isolation and under conditions not representative of normal dietary exposure. EFSA noted the extensive first-pass metabolism of dietary glutamate and the lack of evidence for systemic toxicity at dietary intake levels, particularly when glutamate is consumed as part of complex foods rather than as a single compound. Against this background, the subsequent derivation of a group ADI of 30 mg/kg body weight, based on a conservative safety factor applied to animal neurotoxicity data, represents a methodological choice rather than a biologically compelled conclusion.

Earlier evaluations by JECFA and by the former EU Scientific Committee on Food (SCF) had, in contrast, allocated a group ADI “not specified” for L-glutamic acid and its ammonium, calcium, magnesium, monosodium, and potassium salts [38,39,40]. In its review of amino acids used as flavouring agents, JECFA concluded that the L-forms of the evaluated α-amino acids are macronutrients and normal components of protein, and that their use as flavouring agents at estimated current intakes would not raise any safety concerns; accordingly, the Committee maintained the ADI “not specified” for L-glutamic acid and its salts. Similarly, the SCF considered glutamate a normal dietary constituent and saw no safety concern at the then-current use levels, leading it also to assign an ADI “not specified”. Notably, no substantial new pivotal toxicological evidence became available between these assessments and EFSA’s 2017 re-evaluation that would, in itself, warrant the markedly more conservative numerical group ADI of 30 mg/kg bw per day, indicating that differences in risk-assessment approach rather than in the underlying data largely explain the divergence between these committee conclusions.

Roberts et al. argued that this ADI is overly conservative and not scientifically justified, given human metabolic handling, the dietary matrix, and the long history of safe consumption of glutamate-containing foods, concluding that glutamate “behaves as a safe nutrient” rather than a conventional additive [9]. Similar conclusions were reached by the Norwegian Scientific Committee for Food Safety (VKM), which reviewed the EFSA assessment and emphasised that glutamate exposure from habitual diets, including infant nutrition, does not raise safety concerns when biological context and metabolic capacity are considered, and that guidance values falling below normal intakes risk being misinterpreted as indicating danger [41].

Taken together, the examples of vitamin C, iodine, and glutamate illustrate three fundamentally different ways in which nutrients and nutrient-like substances challenge the classical toxicological risk assessment paradigm. They demonstrate that while the NOAEL/BMD-uncertainty factor approach remains indispensable for many food additives, its uncritical application to nutrients can lead to outcomes that are scientifically unsound, nutritionally inappropriate, or both. These cases reinforce the need for a context-specific risk assessment framework that integrates toxicology with human physiology, nutrition science, and realistic exposure assessment.

## 4. Glutamate Concentrations in Human Milk: Empirical Exposure Data and Biological Interpretation

Multiple independent studies have consistently demonstrated that free glutamate is the most abundant free amino acid in human milk and that its concentration increases over the course of lactation, leading to substantial daily intakes in exclusively breastfed infants. Longitudinal and cross-sectional analyses across diverse populations indicate that free glutamate typically accounts for more than half of the total free amino acid pool in human milk, with mean concentrations rising from approximately 1.2 mM in early lactation to around 1.7–1.8 mM in mature milk [42,43,44]. These patterns are globally consistent and appear to be tightly regulated, suggesting a physiological role rather than random variation [45]. Metabolomic studies further show that free glutamate and glutamine increase while many other metabolites decline or stabilise over time, and that free glutamate is produced within the mammary gland from other amino acids, implying an energetic investment by the mother in maintaining these levels [46,47,48].

Quantitative intake estimates derived from these concentration data indicate that a typical exclusively breastfed infant ingests mean free glutamate amounts of about 35–40 mg/kg body weight per day, with upper-range intakes reaching 70–135 mg/kg body weight per day when both milk volume and milk glutamate concentrations are at the high end of the normal range [49]. These values refer specifically to free glutamate and are distinct from total glutamate intake, which includes protein-bound glutamate and therefore is substantially higher. Corresponding values for infant formula are summarised in Table 1.

Thus, free glutamate intake levels in breastfed infants by far exceed the EFSA group ADI of 30 mg/kg body weight, which is derived from glutamic acid and its salts. In solutions, these salts dissociate to yield glutamate in the same ionic form as naturally occurring free glutamate, yet such intake levels occur during exclusive breastfeeding, which is widely regarded as safe and beneficial. In addition, metabolic studies show that ingested glutamate in infants is almost completely metabolised by the intestinal mucosa and liver, with only minimal amounts reaching the systemic circulation, and its passage across the blood–brain barrier is tightly restricted [36]. Together, these factors greatly limit any potential neurotoxic exposure [49,50]. A similar pattern is observed in adults, where extensive first-pass intestinal metabolism and limited brain uptake similarly prevent substantial central nervous system exposure to dietary glutamate, even at high intakes [35].

**Table 1:** Free and total glutamate concentrations in human milk and infant formula, including the estimated intakes in infants in the first 6 months of life.

	Free Glutamate			Total Glutamate			Source
	Concentration mM	Estimate Mean Intake mg/kg Bodyweight/Day	Estimated Upper Range Intake mg/kg Bodyweight/Day	Concentration mM	Estimate Mean Intake mg/kg Bodyweight/Day	Estimated Upper Range Intake mg/kg Bodyweight/Day	
Human milk (term, mature; exclusively breastfed)	~1–2	~35–40	Up to 135	~13–15	~300	Up to 380	[42,43,49]
Cow’s milk formula (CMF, intact protein, sole feed)	<0.1	~0.7	Up to 1	~18	~390	Up to >450 High consumers 0.5–3.5 months	[49,51]
Extensively hydrolysed formula (sole feed)	~2–6 Brand dependent	~172	Up to 250	~30	~650	Up to 750 High consumers 0.5–3.5 months	[49,51]
Amino acid-based formula (sole feed)	Total level varies with formulation, often in the same order as or higher than CMF. It is in some formula replaced by glutamine	~0–600 not routinely reported					[52] and product-specific specification

To place these breast-milk exposures in a physiological and regulatory context, it is informative to consider free glutamate intakes in infants who are not breastfed but receive formula as their sole or main source of nutrition, including products whose composition and safety have been evaluated within the EFSA framework. Standard CMF-based formulas contain substantially lower concentrations of free glutamate than human milk, resulting in markedly lower free-glutamate intakes (typically well below 5 mg/kg body weight per day in early life). In contrast, extensively hydrolysed protein formulas and some amino acid–based formulas contain considerably higher free glutamate concentrations than both human milk and standard formulas, leading to intakes that can approach or exceed those of breastfed infants (often >100 mg/kg body weight per day in young infants), without any indication of an adverse effect. Across all feeding modes, however, total glutamate exposure (free plus protein bound) is much higher than free glutamate exposure alone, with typical intakes in the order of ~300–750 mg/kg body weight per day during the first months of life. These data underscore that free glutamate represents, for human milk and CMF with intact protein, a fraction of overall glutamate exposure, whereas in extensively hydrolysed formulas and amino acid-based formulas, most or all of the glutamate is present in the free form.

Infants that are breastmilk-fed routinely exceed the EFSA group ADI for free glutamate without any evidence of adverse effects, supporting the notion that additive-based ADIs are not biologically appropriate reference values for this nutrient in infants. These findings reinforce earlier conclusions that setting an ADI below the range of normal, safe intake in infants is inappropriate, e.g., when applied to glutamate. Together, empirical data on glutamate concentrations in human milk and infant intake provide compelling evidence that exceedance of additive-based ADIs does not equate to risk in the context of infant nutrition. Rather, these data highlight the necessity of interpreting exposure within a biological and developmental framework, using breast milk as the appropriate reference for safe and adequate intake.

## 5. Glutamate in Early Life: Physiological Functions and Potential Health Benefits

The exposure data above show that exclusively breastfed infants routinely ingest about 35–40 mg/kg body weight per day of free glutamate, with upper intakes up to roughly 70–135 mg/kg body weight per day, exceeding the current EFSA group ADI of 30 mg/kg body weight per day for glutamic acid–glutamates. At the same time, total glutamate intakes (free plus protein bound) from human milk and various formulas are several hundred mg/kg body weight per day, highlighting that these intakes fall within the range of normal infant nutrition and must be interpreted in a physiological rather than purely toxicological framework.

Across this intake range, total glutamate fulfils several key roles: in the gut it acts as a major oxidative fuel for enterocytes and a luminal signal that regulates digestion and satiety; systemically it supports nitrogen metabolism and glutathione synthesis; and in the nervous system it is the main excitatory neurotransmitter and precursor of peripherally produced GABA that contributes to gut–brain and neuroimmune signalling [53,54].

In early life, protein digestion and the release of free glutamate are constrained by immature digestive capacity, which makes the distinction between free and protein-bound glutamate particularly relevant. Gastric pH after feeds is relatively high, while pepsin and pancreatic proteases (trypsin, chymotrypsin) are produced at lower levels than in adults, and a substantial fraction of dietary protein remains as small peptides rather than fully hydrolysed to free amino acids [55,56]. At the same time, the intestinal peptide transporter PepT1 is highly expressed in the neonatal period, and di- and tripeptides are absorbed more efficiently than free amino acids, as demonstrated in preclinical models [57,58]. This means that much of the glutamate derived from dietary proteins is taken up as part of peptides and then released intracellularly within enterocytes, rather than appearing as free glutamate in the lumen. Consequently:

- Free glutamate present in human milk or formulas is immediately available in the lumen to activate taste and calcium-sensing receptors and to act as a local signal for digestion and satiety, without requiring proteolysis [59,60,61].
- Total glutamate (free plus that released from partially digested protein and absorbed as peptides) provides the main quantitative source of glutamate for enterocyte metabolism [61].

In other words, current evidence suggests that immature digestion in early life limits the appearance of newly liberated free glutamate in the lumen from protein, which increases the relative importance of ingested free glutamate for luminal signalling, while the combination of free and peptide-derived glutamate sustains the very high oxidative and metabolic demands of the intestinal mucosa.

### 5.1. Functions Primary Driven by Free Glutamate

In the intestinal lumen, free glutamate is recognised by several receptors, including umami receptors such as T1R1/T1R3 and the calcium-sensing receptor, which are expressed in the stomach and small intestine and can contribute to stimulation of gastric acid and pepsin secretion, as well as to the release of hormones such as gastrin, cholecystokinin, glucagon-like peptide-1 (GLP1), peptide YY, and indirectly, somatostatin. These hormonal and neural signals influence digestion, gastric emptying, motility, and satiation, and are transmitted to the central nervous system via vagal pathways, thereby participating in the early phases of the gut–brain axis [54,62].

Free glutamate also exerts direct effects on feeding behaviour and growth regulation. Experimental work in infants indicates that increasing the free glutamate content of CMF to 5.7 mM leads to lower intake at a meal and earlier satiation, without increasing subsequent hunger or intake, and similar effects are observed when infants are fed extensively hydrolysed formulas themselves [63]. Consistent with this, casein-based extensively hydrolysed formulas, which contain high levels of free glutamate, are associated with lower weight-for-age and BMI-for-age Z scores, as well as slower weight gain, compared with standard formulas, bringing growth trajectories in the first year of life closer to those of breastfed infants [64,65,66]. In human milk, higher free glutamate concentrations (up to 2 mM) in the first months of life correlate positively with early weight and length gain [67,68], suggesting that free glutamate supports adaptive growth in breastfed infants, and it may also help moderate excessive gain in formula-fed infants.

Free glutamate also serves as a key substrate for microbial production of γ-amino-butyric acid (GABA) via bacterial glutamate decarboxylase systems. GABA produced in the gut is increasingly recognised as a mediator of enteric nervous system signalling, gut motility, and local neuroimmune communication, with emerging evidence, from preclinical models and observational human studies, linking gut microbial GABA pathways to early life neurodevelopment [69].

### 5.2. Function Driven by Total Glutamate

Many metabolic roles of glutamate depend on the total supply of glutamate (free plus that released from dietary proteins in human milk or formula). In neonates, intestinal epithelial cells exhibit high protein turnover and rely heavily on glutamate, glutamine, and aspartate as primary oxidative fuels; roughly 70–80% of enteral glutamate is extracted by splanchnic tissues and largely oxidised to carbon dioxide or incorporated into alanine, proline, and glutamine [53]. This sustained oxidation, which depends on the combined pool of free and protein-derived glutamate, supports intestinal energy metabolism, villus growth, mucosal thickness, and tight junction integrity, thereby strengthening barrier function [49,70].

Systemically, total glutamate intake underpins glutamate’s role as a central hub in nitrogen metabolism and amino acid interconversion, including trans-deamination with glutamine, proline, histidine, arginine, and ornithine, and entry into the TCA cycle as α-ketoglutarate. Both free and protein-bound glutamate contribute to glutathione synthesis and polyglutamated folate formation, processes that are critical for antioxidant defence, redox homeostasis, and one-carbon metabolism during rapid growth. Experimental data in neonatal and juvenile animal models indicate that intestinal glutathione pools have a short half-life and depend heavily on ongoing luminal glutamate and related amino acid supply to maintain redox balance and support epithelial proliferation [4,71,72].

### 5.3. Integrating Functions with High Intake and ADI Considerations

Overall, receptor-mediated signalling in the gut lumen, satiety responses, and certain growth effects are primarily driven by free glutamate. In contrast, the high total glutamate intake from human milk and infant formulas (free plus protein-bound) supports intestinal energy metabolism, nitrogen handling, glutathione synthesis, and other systemic metabolic pathways. At the same time, brain glutamate homeostasis is largely maintained independently of dietary intake through local synthesis and recycling within the central nervous system. As a result, normal variations, and even substantial increases, in dietary glutamate intake do not translate into proportionally higher brain glutamate exposure. In this context, the high free and total glutamate intakes characteristic of early life appear to be a normal and physiologically purposeful feature of infant nutrition rather than an indicator of risk, which has important implications for how ADI-style guidance values are interpreted in infants (see Section 8).

## 6. Example: Human Milk Oligosaccharides and the Limits of Toxicology-Driven Novel Food Assessment

Human milk oligosaccharides (HMOs) provide a further, highly instructive example of the limitations of applying classical toxicological risk assessment approaches to substances that are physiologically relevant and part of the normal human diet at specific life stages. HMOs are complex, non-digestible carbohydrates that occur naturally and abundantly in human breast milk, where they represent the third most abundant solid component after lactose and lipids. Concentrations in mature human milk are typically about 5–25 g/L, so that a fully breastfed infant ingests on the order of 5–20 g HMOs per day, depending on lactation stage and maternal HMO phenotype [73,74].

From a regulatory perspective, isolated HMOs produced via fermentation or enzymatic synthesis have been evaluated in the European Union under the Novel Food Regulation, as they were not consumed as isolated ingredients to a significant degree prior to 1997. As such, they have been subject to EFSA Novel Food safety assessments, which rely on principles derived from toxicological risk assessment, including hazard identification, exposure estimation, and margin-of-safety considerations.

The application of a classical toxicological paradigm to HMOs, however, again presents conceptual challenges from the outset. HMOs are non-digestible, largely unabsorbed in the small intestine, and reach the colon intact, where they serve as selective substrates for specific members of the gut microbiota, particularly *Bifidobacterium* species [75,76]. Their biological effects are initiated locally and are largely microbiota-mediated, with downstream systemic consequences for immune development and regulation arising from microbial metabolites such as short-chain fatty acids (SCFA), rather than from direct systemic exposure to intact HMOs [76]. At the same time, a small but measurable fraction of intact HMOs can be detected in the circulation and urine of breastfed infants, and in infants receiving HMO-supplemented formula, indicating that systemic exposure does occur at low levels and is therefore taken into account in safety evaluations. The consumption of HMOs by infants has been shown to have a number of beneficial effects on the developing gut microbiome and immune system, as well as on overall health in early life [77,78]. Traditional toxicological endpoints such as organ toxicity, genotoxicity, or carcinogenicity are neither expected nor biologically plausible.

In EFSA’s Novel Food evaluations of individual HMOs such as lacto-N-neotetraose (LNnT) and 2′-fucosyllactose (2′-FL) [79,80], the safety of these ingredients has therefore been assessed using a weight-of-evidence approach that departs substantially from the standard NOAEL/BMD-uncertainty factor paradigm. Key elements include comparison with naturally occurring exposure in breastfed infants, compositional and structural equivalence to HMOs in human milk, toxicokinetic data showing minimal systemic exposure and renal excretion of the absorbed fraction, and lack of adverse effects in sub-chronic toxicity studies even at very high doses. In several cases, no adverse effects were identified at the highest doses tested, resulting in reference points that exceed realistic dietary exposures by large margins [79,80]. Safety considerations also extend to potentially sensitive subpopulations, including infants with allergy or an atopic predisposition, by evaluating clinical trial data, patterns of adverse events, and, where relevant, guidance on allergenicity and immunological effects. To date, these assessments have not identified specific safety concerns for authorised HMOs under the proposed conditions of use.

A particularly revealing aspect of these assessments is that the target population for novel food HMOs often includes infants, a group traditionally considered highly sensitive in toxicological risk assessment. Under a classical additive framework, this would normally trigger conservative assumptions and large UFs. Instead, EFSA evaluations explicitly recognise that infants are already exposed to substantially higher amounts of HMOs through breastfeeding than would result from consumption of HMO-containing infant formula or other foods. Methodologically, anticipated intakes from the proposed uses (expressed on a mg/kg body weight basis) are systematically compared with the distribution of HMO intakes in exclusively breastfed infants, using human milk concentration data and high-percentile consumption scenarios, so that even high consumers of HMO-containing products are unlikely to exceed the upper range of physiologically normal exposure. As a result, exposure comparisons with breastfed infants play a central role in risk characterisation, effectively replacing default UFs with biological contextualization.

If a classical food-additive approach were applied rigidly, requiring identification of a NOAEL from animal studies and application of a default 100-fold UF, the resulting “safe” intake levels for HMOs would likely fall below, and far below, intakes that are normal and beneficial in breastfed infants. Such an outcome would contradict both evolutionary biology and empirical human experience and would undermine the rationale for using HMOs in infant nutrition altogether. The HMO example therefore illustrates a further distinct limitation of the classical paradigm: when a substance is a normal and abundant component of human breast milk, toxicological risk assessment cannot be meaningfully decoupled from developmental biology and comparative human exposure. In such cases, safety is demonstrated not by identifying ever-lower thresholds of adversity, but by demonstrating equivalence to natural exposure patterns, absence of systemic toxicity, and biological plausibility of predominantly local effects.

## 7. Comparative Overview: Four Ways Nutrients Challenge Classical Risk Assessment

**Figure 2:**
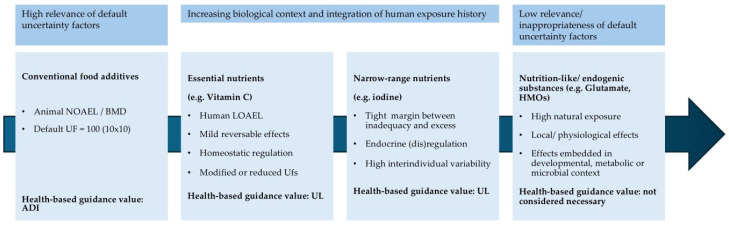
The scheme illustrates the decreasing suitability of the classical NOAEL/BMD–uncertainty factor paradigm across different categories of food ingredients. While default UFs are appropriate for conventional food additives, their relevance diminishes for nutrients and nutrient-like substances, where biological function, human exposure history, and physiological regulation become dominant determinants of risk characterisation.

Taken together, the examples of vitamin C, iodine, glutamate, and human milk oligosaccharides illustrate four essentially different ways in which nutrients and nutrient-like substances challenge the classical toxicological risk assessment paradigm (Table 2). Vitamin C demonstrates how large default UFs become inappropriate when adverse effects are mild, local, and reversible. Iodine highlights the difficulty of managing nutrients with a narrow safe intake range governed by endocrine regulation and population heterogeneity. Glutamate shows how high background exposure and extensive first-pass metabolism can render the derivation of an ADI biologically implausible. Human milk oligosaccharides, finally, illustrate the limits of toxicology-driven assessment when a substance is a natural, developmentally relevant component of the human diet, consumed at high levels by the very young, i.e., a population group traditionally considered most vulnerable. This overall pattern is summarised schematically in Figure 2, which illustrates how the suitability of the classical NOAEL/BMD–uncertainty factor paradigm decreases from conventional food additives to nutrients and nutrient-like substances.

Collectively, these cases reinforce that while the NOAEL/BMD-uncertainty factor framework remains a cornerstone of food additive risk assessment, it must be applied with flexibility and scientific judgement when addressing nutrients, nutrient-like substances, and novel foods with established physiological roles. Effective risk assessment in these contexts requires integration of toxicology with human biology, nutrition science, developmental considerations, and realistic exposure patterns, ensuring that health-based guidance values remain both protective and biologically meaningful.

## 8. Amino Acids in Infant Formula: Biological Context and Regulatory Acceptance

Infant formula provides a biologically relevant context in which the limitations of classical ADI-based risk assessment become particularly evident. Amino acids, including glutamate, are not only intrinsic components of human milk but are also present in infant formulas in both protein-bound and free form.

This is most clearly illustrated by the use of protein hydrolysates and amino acid-based formulas. Enzymatic hydrolysis of proteins in partially and extensively hydrolysed formulas results in substantially increased concentrations of free amino acids compared with intact-protein formulas, and in some cases compared with human milk. Amino acid–based formulas, used for specific medical indications, represent the extreme of this continuum, with all nitrogen provided in free amino acid form. In both cases, dietary exposure to free glutamate is high by any conventional exposure metric.

Despite this, the European Food Safety Authority (EFSA) has consistently concluded that authorised hydrolysed and amino acid-based formulas are safe and nutritionally appropriate when used as intended. These conclusions are not derived from the application of default UFs or the establishment of additive-style ADIs. Instead, EFSA’s evaluations are grounded in nutritional adequacy, amino acid balance, metabolic capacity, clinical growth outcomes, and the absence of adverse effects. Extensive clinical data demonstrate normal growth, appropriate nitrogen utilisation, and good tolerance, even under conditions of high free amino acid intake. From a toxicological perspective, these regulatory outcomes are instructive. If free glutamate were assessed in isolation using a conventional NOAEL–uncertainty factor framework, the resulting HBGVs would be exceeded by infants consuming hydrolysed or amino acid–based formulas. The regulatory acceptance of such products therefore indicates that, for glutamate in infant nutrition, risk assessment must be aligned with physiological handling and clinical outcomes rather than with generic exposure cut-offs. Importantly, EFSA, in its evaluations of infant formula, recognises amino acids, including glutamate, as normal dietary constituents with essential or conditionally essential functions in growth and development. Safety assessment therefore focuses on whether intakes support normal physiology without causing imbalance or adverse effects, rather than on identifying toxicological thresholds. This reflects a broader principle: substances that are integral to normal metabolism and consumed at gram-per-day levels in early life cannot be meaningfully assessed using hazard-based models designed for exogenous chemicals with no nutritional role. In this light, infant formula, particularly hydrolysed and amino acid–based products, serves as a compelling real-world example demonstrating that high dietary exposure to free glutamate is not inherently hazardous. Rather, it represents a physiologically purposeful and well-regulated aspect of infant nutrition. Accordingly, the regulatory treatment of glutamate in infant formula underscores the limited relevance of ADI-based frameworks for nutrient-like and endogenously handled substances and highlights the need for risk assessment approaches that are anchored in a biological context rather than abstract exposure limits.

## 9. Discussion

The deliberately provocative title of this paper, “Glutamate: safe and adequate intake levels for infants—should breast milk be taken off the market?”, highlights a core methodological challenge in contemporary food safety assessment. When classical toxicological paradigms developed for food additives are applied uncritically to nutrients and biologically intrinsic food components, they can yield conclusions that are not only biologically implausible but also inconsistent with established nutritional science and regulatory practice [3]. It is therefore notable that both JECFA and the former SCF, evaluating essentially the same toxicological evidence base, had previously allocated a group ADI “not specified” to glutamate and its salts, whereas EFSA’s 2017 re-evaluation introduced a numerical group ADI of 30 mg/kg bw per day, underscoring how different applications of the same paradigm can lead to divergent regulatory outcomes [38,39,40].

These discrepancies should be interpreted against the background of EFSA’s mandate as a risk assessment body, working within a precautionary framework for food additives and providing scientific advice to risk managers, rather than as evidence of disregard for nutritional science. The concern expressed here is directed at the suitability of this framework when applied to nutrient-like substances, not at the scientific integrity of EFSA’s evaluations.

Figure 2 schematically summarises this tension between toxicity-driven and context-driven assessment for the examples discussed in this Viewpoint. Vitamin C occupies a position where classical toxicological endpoints are mild and reversible, so that large default uncertainty factors can produce reference values that are difficult to reconcile with nutritional adequacy. Iodine exemplifies a narrow ‘safe range’, where both deficiency and excess carry risks and where risk characterisation must account for background intake and population variability. Glutamate illustrates a nutrient-like metabolic substrate with high habitual intakes and extensive first-pass metabolism, for which a numerically restrictive ADI can fall below normal exposures without indicating a realistic safety concern. HMOs finally represent a case in which safety is assessed primarily by comparison with physiologically normal exposures in breastfed infants rather than by deriving an additive-style ADI. Together, these placements in Figure 2 highlight why an integrative, physiology-anchored framework is needed when evaluating nutrients and nutrient-like substances, particularly in infant nutrition.

The examples discussed throughout this paper converge on a single, coherent conclusion. Breast milk contains substantial amounts of free glutamate and HMOs, resulting in infant intakes that may exceed health-based guidance values derived using default UFs. Yet breast milk has an unparalleled history of safe use (!) and is widely recognised as the reference standard for infant nutrition. EFSA Novel Food evaluations of isolated HMOs explicitly use exposure from breast milk as a benchmark for safety, replacing default additive-style assumptions with contextualisation against physiologically normal ranges of intake [83].

This logic is further reinforced by EU regulatory decisions on infant formula, informed by EFSA’s scientific opinions on the essential composition of infant and follow-on formulae and on formulae manufactured from protein hydrolysates, together with Commission Delegated Regulation (EU) 2016/127 [84,85,86].

Protein hydrolysate formulas, which result in even higher intakes of free amino acids, particularly glutamate, have likewise been approved following demonstration of normal growth, absence of adverse clinical effects, and long-standing safe use in infants, including vulnerable subpopulations.

Taken together, breast milk, standard infant formula, amino-acid-supplemented formula, and protein hydrolysates all lead to infant exposures that would be difficult or impossible to reconcile with a rigid application of classical NOAEL/BMD–uncertainty factor frameworks. Rather than indicating a safety concern, this apparent inconsistency illustrates the limits of extrapolating additive-based toxicological models to substances that are intrinsic to human metabolism, development, and nutrition, and whose safe exposure ranges are defined by normal physiology.

The answer to the question posed in the title is therefore unequivocal: breast milk should not, and cannot, be considered unsafe on the basis of glutamate or HMOs. On the contrary, these components exemplify why infant risk assessment must be grounded in biological function, developmental context, human exposure history, and nutritional adequacy. Current EFSA guidance on substances in infant foods, together with EFSA opinions on glutamate and HMOs, implicitly acknowledges that health-based guidance values for the general population cannot be applied mechanically to early infancy without such contextualisation [87,88]. Viewed through a risk–benefit lens, the central question is therefore not only whether high-end intakes of these components might pose theoretical risks, but whether restricting them would simultaneously erode well-documented physiological and developmental benefits, thereby worsening the overall health balance for infants.

Ultimately, this analysis underscores the need for proportional, context-driven application of toxicological principles. For infants in particular, health-based guidance values must protect against genuine risk without undermining biologically normal and demonstrably safe patterns of exposure. Only by integrating toxicology with nutrition and human physiology can risk assessment remain both scientifically credible and aligned with public health objectives.

## 10. Conclusions

This paper demonstrates that classical toxicological risk assessment approaches, while indispensable for conventional food additives, are not universally applicable to nutrients and biologically intrinsic food components, particularly in the context of infant nutrition. Through the examples of glutamate, human milk oligosaccharides, amino acids, and protein hydrolysates, it is evident that reliance on default UFs and additive-style acceptable daily intakes can produce outcomes that are biologically implausible and inconsistent with established regulatory practice.

EFSA’s own assessments across nutrients, novel foods, and infant formula ingredients consistently apply a context-driven approach, integrating human exposure history, physiological function, developmental biology, and nutritional adequacy. These assessments implicitly recognise that exposure levels exceeding additive-based thresholds do not constitute a safety concern when they reflect physiologically normal dietary patterns and are supported by extensive evidence of safe use.

The conclusion is therefore not that toxicological principles should be abandoned, but that they must be applied proportionally and with scientific and regulatory judgement. For infants in particular, health-based guidance values should protect against genuine adverse effects without contradicting the biological reality of breast milk and authorised infant formulas. Risk assessment frameworks that fail this test risk undermining both scientific credibility and public health protection and messaging.

In this context, breast milk remains the appropriate biological reference for infant nutrition, and its composition, including glutamate and HMOs, should inform and anchor, rather than conflict with, regulatory safety assessments.

## Figures and Tables

**Table 2:** Key characteristics of vitamin C, iodine, glutamate, and HMOs relevant to classical toxicological risk assessment, illustrating how physiological role, intake range, critical effects, and data sources influence the applicability of the NOAEL/BMD–uncertainty factor paradigm.

Aspect	Vitamin C	Iodine	Glutamate	HMOs	Source
Nutrient	Yes	Yes	Yes	Yes	
Normal dietary constituent	Yes	Yes	Yes (both as free glutamate and as part of protein)	Yes (in breastmilk for infants)	
Food additive	E300 (ascorbic acid)		E620 through E625 (glutamate and its related salts)		
Regulatory category outside dietary constituent	Food additive		Food additive	Novel food	
Physiological role	Antioxidant, cofactor	Thyroid hormone synthesis	Metabolic substrate, neurotransmitter precursor	Microbiota modulation, immune development	[22,23,27,28,31,33,34,53,54,75,77,78]
Typical intake range	~50–300 mg/day	~100–300 µg/day	Total intake ~5–20 g/day	Infants: 5–20 g/day (via breast milk)	[74,81,82]
Critical adverse effect	Diarrhoea	Thyroid dysfunction	None at dietary intake level	None identified	
Nature of effect	Mild, local, reversible	Functional endocrine dysregulation	Not toxicologically relevant	Local, microbiota-mediated	
Key data source	Human intervention studies	Human observational & intervention data	Human kinetic & dietary data	Human milk composition + toxicology	
Point of departure	LOAEL ≈ 3 g/day	Thyroid effects ≈ 1.7–1.8 mg/day	High-dose animal studies (poorly relevant)	No adverse effects at highest doses	
Health-based guidance value (EFSA)	UL = 2 g/day (US IOM) (EFSA: no UL identified)	UL = 600 µg/day	ADI 30 mg/kg-bw (may fall below normal intake)	Intake justified by equivalence to breast milk	[26,37,79,80]
UFs (currently used by EFSA)	-	~3	100	Replaced by biological contextualisation	
Core paradigm limitation	Default UFs for deriving an ADI undermine nutritional adequacy	Narrow safe range between too high and too low intake	Default UFs for deriving an ADI undermine nutritional adequacy	Developmental biology outweighs toxicology	

## Data Availability

No new data were created or analysed in this study.

## Abbreviations

The following abbreviations are used in this manuscript:

ADI	Acceptable Daily Intake
BMD	Benchmark Dose
BMDL	Benchmark Dose Lower Confidence Limit
CMF	Cow’s Milk Formula
EFSA	European Food Safety Authority
FAO	Food and Agriculture Organization of the United Nations
GLP-1	Glucagon-Like Peptide-1
HBGV(s)	Health-Based Guidance Value(s)
HMOs	Human Milk Oligosaccharides
JECFA	Joint FAO/WHO Expert Committee on Food Additives
LNnT	Lacto-N-neotetraose
NDA Panel	EFSA Panel on Dietetic Products, Nutrition and Allergies
NOAEL	No-Observed-Adverse-Effect Level
PEPT1	Peptide Transporter 1
SCFA	Short-Chain Fatty Acid
TCA cycle	Tricarboxylic Acid Cycle
TSH	Thyroid-Stimulating Hormone
UF(s)	Uncertainty Factor(s)
UL(s)	Tolerable Upper Intake Level(s)
VKM	Norwegian Scientific Committee for Food Safety
WHO	World Health Organization
